# Burden and Factors Associated With Depressive Symptoms, Anxiety Symptoms, and Probable Post‐Traumatic Stress Disorder in Eastern Shan State, Myanmar: An Analytical Cross‐Sectional Study

**DOI:** 10.1002/hsr2.72683

**Published:** 2026-06-19

**Authors:** Thein Myint Twin, Kyaw Min Htike, Kaung Myat Soe, Roshan Kumar Mahato

**Affiliations:** ^1^ Faculty of Public Health Khon Kaen University Khon Kaen Thailand

**Keywords:** anxiety, depression, mental health, Myanmar, PTSD

## Abstract

**Background and Aims:**

Eastern Shan State, particularly Kengtung, Tachileik, and Mong Hsat, has recently faced ongoing political and socioeconomic challenges. However, limited data exist on mental health impacts in this region. This study aimed to determine the prevalence of depressive symptoms, anxiety symptoms, and probable post‐traumatic stress disorder (PTSD) and identify associated factors among individuals living in Eastern Shan State of Myanmar.

**Methods:**

An analytical cross‐sectional study was conducted among 448 adults (March–July 2025) using the Center for Epidemiologic Studies Depression Scale (CES‐D‐10), Generalized Anxiety Disorder scale (GAD‐7), Short Post‐Traumatic Stress Disorder Rating Interview (SPRINT), and Multidimensional Scale of Perceived Social Support (MSPSS) in Shan State of Myanmar. Generalized Linear Mixed Models (GLMM) with random effects for household clustering were applied to identify potential risk factors.

**Results:**

The prevalence of depressive symptoms, anxiety symptoms, and probable PTSD was 46.4% (208/448; 95% CI: 41.8–51.1), 19.6% (88/448; 95% CI: 16.2–23.6), and 9.4% (42/448; 95% CI: 7.0–12.5), respectively. Depressive symptoms were associated with unemployment (AOR: 1.79; 95% CI: 1.14–2.80), inadequate financial status (AOR: 1.75; 95% CI: 1.16–2.64), food insecurity (AOR: 2.25; 95% CI: 1.05–4.83), low perceived social support from a special person (AOR: 3.23; 95% CI: 2.00–5.21), and forced displacement (AOR: 2.24; 95% CI: 1.13–4.40). Similarly, anxiety symptoms were associated with food insecurity (AOR: 4.33; 95% CI: 1.99–9.41), low special person support (AOR: 2.94; 95% CI: 1.68–5.13), nonfamily living arrangements (AOR: 3.54; 95% CI: 1.94–6.47), and forced displacement (AOR: 3.21; 95% CI: 1.61–6.41). Furthermore, probable PTSD was associated with living without family (AOR: 5.65, 95% CI: 2.80–11.39) and forced displacement (AOR: 4.98, 95% CI: 2.31–10.76).

**Conclusion:**

This study contributes to the limited empirical evidence on mental health in conflict‐affected regions of Myanmar, where structural vulnerabilities such as economic hardship, displacement, and social isolation amplify psychological distress. The results highlight the urgent need for integrated interventions combining psychosocial support, livelihood recovery, food security, and community‐based networks to strengthen resilience in fragile settings.

## 1 Introduction

Mental health disorders such as depression, anxiety, and post‐traumatic stress disorder (PTSD) are significant public health concerns globally [1]. It is estimated that one in five people experience these conditions, with varying severity; around 9% develop moderate to severe symptoms often requiring clinical intervention. In Myanmar, a study found that 61% of adults reported probable depression and 58% reported probable anxiety, indicating a substantial mental health burden [2].

In the Asia‐Pacific region, the burden of mental health disorders is substantial. A systematic review of populations in Southeast Asia reported pooled prevalences of 28.54% for depression, 29.94% for PTSD, and 31.11% for anxiety [3]. Comorbid conditions are common, and studies show that depression, anxiety, and PTSD often overlap in affected populations [4]. Regional studies reported variable prevalence estimates; for example, PTSD prevalence was 9.5% in Thailand and 71% in India highlighting the clinical significance of these mental health issues [5].

Recent studies have highlighted the significant impact of conflict and political instability on mental health in Myanmar. A nationwide study found that factors such as unemployment, inadequate financial status, food insecurity, low perceived social support, and forced displacement were identified as significant risk factors for depression [2]. Additionally, a study focusing on adolescent girls and young women in Myanmar reported high levels of depressive symptoms, with exposure to conflict‐related stressors such as displacement, family separation, and restricted access to education being significant contributors [6]. These findings underscore the need for targeted mental health interventions in conflict‐affected regions. Despite the recognized burden, limited empirical data exist on the prevalence and factors associated with depressive symptoms, anxiety symptoms, and probable PTSD in Myanmar, particularly in Eastern Shan State where communities face compounded vulnerabilities including political instability, poverty, displacement, and social marginalization.

**Figure 1:**
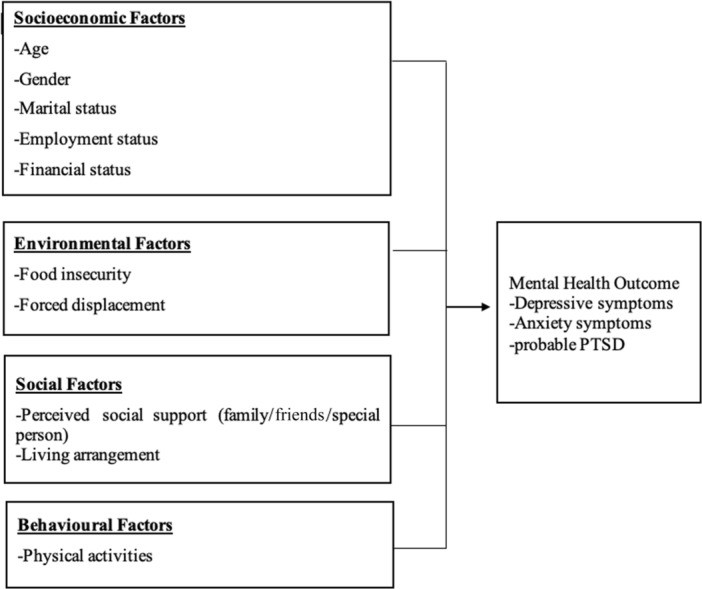
Conceptual framework illustrating the hypothesized relationships between socioeconomic, environmental, and social factors and mental health outcomes among study participants. Socioeconomic factors include employment status and financial status; environmental factors include food insecurity and forced displacement; social factors include perceived social support (from family, friends, and significant others) and living arrangement. Arrows indicate the direction of hypothesized associations with mental health outcomes, including depressive symptoms, anxiety symptoms, and probable PTSD.

Social, economic, and environmental determinants play a critical role in shaping mental health outcomes, particularly in conflict‐affected settings [7]. Food insecurity has been consistently linked to psychological distress, as uncertainty in accessing adequate food acts as a chronic stressor and reflects broader socioeconomic deprivation [8]. Similarly, forced displacement exposes individuals to traumatic events, loss of livelihoods, and disruption of social networks, all of which significantly increase the risk of depression, anxiety, and PTSD [9]. Social support, especially from family and close relationships, serves as a key protective factor by buffering stress and promoting emotional resilience. In fragile contexts such as Eastern Shan State, where communities experience political instability, economic hardship, and social disruption, these factors are particularly relevant and may interact to exacerbate mental health vulnerabilities. Therefore, examining these variables is essential to better understand the underlying factors associated with mental health in this setting. Eastern Shan State has experienced prolonged political instability, armed conflict, and population displacement, contributing to widespread socioeconomic hardship and limited access to healthcare and social services. These contextual challenges have been identified as key drivers of mental health vulnerability in conflict‐affected settings [2]. While previous studies in Myanmar have examined mental health at national or specific population levels, there remains a lack of empirical data focusing on conflict‐affected and socioeconomically vulnerable regions such as Eastern Shan State. This study specifically addresses this gap by examining the prevalence and associated factors of mental health symptoms within this under‐researched and high‐risk setting. A conceptual framework illustrating the hypothesized relationships between key determinants and mental health outcomes is presented in Figure 1.

## 2 Methods

### 2.1 Study Settings

The study was conducted in Eastern Shan State, Myanmar, one of the country's most ethnically diverse and conflict‐affected regions. Data were collected from three major townships such as Kengtung, Tachileik, and Mong Hsat which represent key districts of Eastern Shan State. These areas include a mix of urban and rural communities, with livelihoods primarily based on agriculture, small‐scale trade, and informal economic activities. The region has experienced ongoing political instability, armed conflict, and population displacement, contributing to socioeconomic challenges such as poverty, food insecurity, and limited access to healthcare and social services. These contextual factors make the area particularly relevant for assessing the prevalence and factors associated with depressive symptoms, anxiety symptoms, and probable PTSD.

### 2.2 Sample Size Calculation and Sampling

A total of 448 participants were recruited. The required sample size was initially estimated at 407 using the logistic regression formula proposed by Hsieh et al. [10], which is appropriate for studies with binary outcomes [10]. This approach was selected as the study aimed not only to estimate prevalence but also to examine associations between exposures and mental health outcomes, informed by previous research conducted in Myanmar [11]. To account for design complexity and potential correlation among predictors, a variance inflation factor (VIF) of 8.33 was applied. The VIF was applied to account for clustering and design effects. The value was derived based on assumed intra‐cluster correlation and average cluster size, consistent with recommendations for clustered sampling designs. An additional 10% was added to account for potential nonresponse, resulting in a final target sample size of 448 participants. A multistage sampling approach was used. First, villages were selected using simple random sampling from the available household listing frame. Within selected villages, households were randomly selected, and one eligible participant per household was interviewed. A total of 448 participants were included in the final analysis. All selected participants met the inclusion criteria and completed the interview. There were no significant exclusions after recruitment.

Inclusion criteria were adults aged 18–59 years who had resided in the area for at least 6 months, were able to understand Myanmar or Shan languages, were capable of providing informed consent, and were able to perform basic activities of daily living independently (e.g., eating, dressing, and mobility). Exclusion criteria included individuals who were physically unable to participate in the interview due to severe illness, cognitive impairment, or functional limitations (e.g., bedridden individuals). Institutionalized populations (e.g., hospitalized patients, military personnel, and prisoners) and those unwilling to participate were also excluded. Individuals with known severe mental disorders (e.g., bipolar disorder), intellectual disability, or substance use disorders were not excluded unless they were unable to participate in the interview due to impaired cognitive or functional capacity.

### 2.3 Operational Definition

Key variables were operationally defined as follows.

#### 2.3.1 Food Insecurity (FIES)

The Food Insecurity Experience Scale (FIES) consists of 8 items assessing access to adequate food over the past 12 months. Scores range from 0 to 8, with higher scores indicating greater food insecurity [12]. In this study, scores were categorized as food secure (0–3) and moderate‐to‐severe food insecurity (≥ 4).

#### 2.3.2 Forced Displacement

Participants were asked whether they had experienced displacement due to conflict or related events within the past 12 months (Yes/No).

#### 2.3.3 Living Arrangement

Living arrangements were categorized as living with family/relatives or nonfamily living. Nonfamily living refers to individuals living alone, with friends, or in other nonfamily settings.

#### 2.3.4 Perceived Social Support

Measured using the Multidimensional Scale of Perceived Social Support (MSPSS), which assesses support from family, friends, and a “special person” [13]. A “special person” was described to participants as someone they feel close to and can rely on for emotional or practical support.

#### 2.3.5 Outcome Variables

Depression, anxiety, and PTSD were assessed using Center for Epidemiologic Studies Depression Scale (CES‐D‐10) [14], Generalized Anxiety Disorder scale (GAD‐7) [15], and Short Post‐Traumatic Stress Disorder Rating Interview (SPRINT) [16], respectively, using standard cut‐off scores.

### 2.4 Data Collection

Data were collected using a structured interviewer‐administered questionnaire comprising sociodemographic, socioeconomic, environmental, and psychosocial variables. Mental health outcomes, including depressive symptoms, anxiety symptoms, and probable PTSD, were assessed using validated instruments: CES‐D‐10, GAD‐7, and SPRINT. A structured questionnaire was used to collect information on key variables, including age, sex, marital status, residence, employment status, financial status, food insecurity, perceived social support, living arrangement, physical activity, and forced displacement. These instruments have been widely validated; in this study, content validity and internal consistency reliability were assessed through expert review and pilot testing. The questionnaire was translated using forward–backward procedures to ensure linguistic and cultural appropriateness. Standard cut‐off values based on previous validation studies were applied. Trained data collectors conducted interviews in private settings to ensure confidentiality and participant comfort. Detailed descriptions of measurement tools, scoring procedures, and reliability assessments are provided in Supporting Material S1.

### 2.5 Data Analysis

Data were analyzed using STATA version 18.5 (College Station, Texas, USA). Data completeness was assessed prior to analysis, and missing data were minimal; therefore, complete case analysis was performed. Given the hierarchical structure of the data, with individuals nested within villages, Generalized Linear Mixed Models (GLMM) were applied to account for clustering at the village level. Random intercept models were specified for villages to capture intra‐cluster correlation. Univariable analyses were first conducted for all potential explanatory variables. Variables with *p* value < 0.25 were selected for inclusion in the multivariable models using a backward elimination approach. This threshold was applied to avoid excluding potentially important variables at the initial stage of model building, as recommended in epidemiological modeling approaches [17]. In addition to statistical criteria, variables of theoretical importance were also considered during model development. Multivariable GLMMs were then fitted for each outcome (depressive symptoms, anxiety symptoms, and probable PTSD symptoms) to identify associated factors. Urban–rural residence and other covariates were included as fixed effects in the models. Adjusted odds ratios (AORs) and 95% confidence intervals (CIs) were reported. Model diagnostics were performed to assess goodness‐of‐fit and multicollinearity. Model fit was evaluated using Akaike Information Criterion (AIC) and Bayesian Information Criterion (BIC), and intraclass correlation coefficients (ICC) were calculated to assess the degree of clustering. Variance inflation factor (VIF < 10) indicated no evidence of significant multicollinearity. All models converged appropriately. Statistical significance was set at *p* value < 0.05. All statistical tests were two‐sided. Statistical analyses and reporting were conducted in accordance with established reporting guidelines for clinical research, including SAMPL recommendations.

## 3 Results

The study included 448 participants, with the majority aged 18–39 years and predominantly female. Most participants were employed and reported inadequate financial status. Detailed characteristics are presented in Table 1.

**Table 1:** Sociodemographic characteristics of the study population (*n* = 448).

Characteristics	Number (*n*)	Percentage (%)
Age		
18–39	332	74.11
40–59	116	25.89
Mean (SD)	32.76 (±10.79)	
Median (Min:Max)	30 (18:59)	
Gender		
Male	186	41.52
Female	262	58.48
Marital status		
Married	252	56.25
Single/divorce/widow	196	43.75
Residence		
Urban	247	55.13
Rural	201	44.87
Current employment status		
Employed	325	72.54
Unemployed	123	27.46
Financial status		
Adequate	191	42.63
Inadequate	257	57.37
Food insecurity (FIES)		
Secure (0–3)	409	91.29
Moderate‐to‐severe food insecurity (FIES ≥ 4)	39	8.71
Perceived social support from family		
Low (< 3)	92	20.54
Moderate (3–5)	165	36.83
High (> 5)	191	42.63
**P**erceived social support from friend		
Low (< 3)	107	23.88
Moderate (3–5)	253	56.47
High (> 5)	88	19.64
Perceived social support from special person		
Low (< 3)	113	25.22
Moderate (3–5)	125	27.90
High (> 5)	210	46.88
Living arrangement		
Family/parents/relative	372	83.04
Friends/work partner/alone/other	76	16.96
Physical activity		
Inactivity (< 600 MET score)	63	14.06
Minimally active (600–3000 MET score)	185	41.29
HEPA active (> 3000 MET score)	200	44.64
Forced displacement		
No	399	89.06
Yes	49	10.94

The prevalence of depressive symptoms, anxiety symptoms, and probable PTSD was 46.4% (208/448; 95% CI: 41.8–51.1), 19.6% (88/448; 95% CI: 16.2–23.6), and 9.4% (42/448; 95% CI: 7.0–12.5), respectively. Depressive symptoms were more common among participants who were unemployed, had inadequate financial status, experienced food insecurity, reported low perceived social support, and had experienced forced displacement. Similar patterns were observed for anxiety symptoms and probable PTSD (Table 2).

**Table 2:** Prevalence of depressive symptoms, anxiety symptoms and probable PTSD by sociodemographic, economic, and social support factors (with 95% CI).

Characteristics	Depressive symptoms % (95% CI)	Anxiety symptoms % (95% CI)	Probable PTSD % (95% CI)
Overall	46.43 (41.84–51.08)	19.64 (16.21–23.59)	9.38 (7.00–12.45)
Age			
18–39	45.78 (40.48–51.19)	20.18 (16.20–24.86)	9.94 (7.15–13.66)
40–59	48.28 (39.31–57.35)	18.10 (12.10–26.20)	7.76 (4.08–14.26)
Gender			
Male	45.70 (38.66–52.92)	18.28 (13.35–24.51)	8.06 (4.91–12.96)
Female	46.95 (40.96–53.02)	20.61 (16.13–25.95)	10.31 (7.16–14.62)
Marital status			
Married	43.25 (37.25–49.46)	15.48 (11.51–20.50)	6.75 (4.23–10.60)
Single/divorce/widow	50.51 (43.53–57.47)	25.00 (19.42–31.55)	12.76 (8.76–18.21)
Residence			
Urban	48.99 (42.78–55.22)	22.67 (17.86–28.33)	10.93 (7.60–15.49)
Rural	43.28 (36.58–50.24)	15.92 (11.48–21.67)	7.46 (4.54–12.02)
Current employment status			
Employed	42.77 (37.48–48.23)	19.38 (15.43–24.06)	8.31 (5.75–11.85)
Unemployed	56.10 (47.20–64.42)	20.33 (14.11–28.38)	12.20 (7.48–19.27)
Financial status			
Adequate	37.74 (32.01–43.84)	16.73 (12.64–21.82)	8.95 (6.01–13.12)
Inadequate	58.12 (50.98–64.92)	23.56 (18.06–30.11)	9.95 (6.43–15.09)
Food insecurity (FIES)			
Secure (0–3)	44.25 (39.50–49.12)	17.11 (13.76–21.09)	8.31 (5.99–11.42)
Moderate‐to‐severe food insecurity (FIES ≥ 4)	69.23 (53.22–81.65)	46.15 (31.32–61.71)	20.51 (10.58–36.00)
Perceived social support from family			
Low (< 3)	68.48 (58.29–77.15)	36.96 (27.72–47.26)	16.30 (10.06–25.33)
Moderate (3–5)	58.18 (50.50–65.48)	21.82 (16.16–28.78)	12.12 (7.95–18.06)
High (> 5)	25.65 (19.95–32.34)	9.42 (6.01–14.48)	3.66 (1.75–7.50)
Perceived social support from friend			
Low (< 3)	51.40 (41.97–60.74)	25.23 (17.89–34.33)	10.28 (5.78–17.64)
Moderate (3–5)	52.17 (46.00–58.28)	21.34 (16.72–26.84)	11.86 (8.41–16.47)
High (> 5)	23.86 (16.09–33.88)	7.95 (3.83–15.79)	1.14 (0.16–7.66)
Perceived social support from special person			
Low (< 3)	69.03 (59.91–76.87)	38.94 (30.38–48.23)	17.70 (11.70–25.88)
Moderate (3–5)	53.60 (44.81–62.17)	16.00 (10.55–23.53)	8.80 (4.93–15.22)
High (> 5)	30.00 (24.17–36.55)	11.43 (7.77–16.50)	5.23 (2.92–9.22)
Living arrangement			
Family/parents/relative	42.74 (37.79–47.84)	13.98 (10.80–17.90)	5.38 (3.49–8.20)
Friends/work partner/alone/other	64.47 (53.12–74.40)	47.37 (36.43–58.57)	28.95 (19.86–40.11)
Physical activity			
Inactivity (< 600 MET score)	47.62 (35.64–59.88)	17.46 (9.93–28.88)	11.11 (5.38–21.56)
Minimally active (600–3000 MET score)	51.35 (44.15–58.50)	26.49 (20.62–33.33)	11.35 (7.51–16.80)
HEPA active (> 3000 MET score)	41.50 (34.86–48.47)	14.00 (9.83–19.55)	7.00 (4.18–11.49)
Forced displacement			
No	43.61 (38.81–48.53)	16.04 (12.75–19.99)	6.52 (4.47–9.41)
Yes	69.39 (55.21–80.65)	48.98 (35.38–62.73)	32.65 (21.04–46.87)

### 3.1 Factors Associated With Depressive Symptoms

In the multivariable logistic regression analysis using a GLMM, several factors were found to be significantly associated with depressive symptoms. Adjusted models included variables that met the inclusion criteria (*p* < 0.25) in univariable analysis including age, sex, socioeconomic variables, and other significant covariates. Unemployed individuals had significantly higher odds of depressive symptoms compared to those employed (AOR = 1.79; 95% CI: 1.14–2.80). Participants with inadequate financial status were also more likely to experience depressive symptoms (AOR = 1.75; 95% CI: 1.16–2.64). Food insecurity was another significant factor: individuals with moderate or severe food insecurity (FIES score ≥ 4) had more than twice the odds of depressive symptoms compared to those who were food secure (AOR = 2.25; 95% CI: 1.05–4.83). Low perceived social support from a special person was strongly associated with depressive symptoms, with affected individuals having over three times the odds of depressive symptoms (AOR = 3.23; 95% CI: 2.00–5.21). Finally, participants who experienced forced displacement were significantly more likely to suffer from depressive symptoms compared to those who were not displaced (AOR = 2.24; 95% CI: 1.13–4.40) (Table 3).

**Table 3:** Prevalence and factors associated with depressive symptoms: GLMM (*n* = 448).

Factors	Total	% of depressive symptoms	COR	95% CI	*p*	AOR	95% CI	*p*
Age					0.643			
18–39	332	45.78	1					
40–59	116	48.28	1.11	0.72–1.69				
Gender					0.794			
Male	186	45.70	1					
Female	262	46.95	1.05	0.72–1.53				
Marital status					0.127			
Married	252	43.25	1					
Single/divorce/widow	196	50.51	1.34	0.92–1.95				
Current employment status					0.012			0.011
Employed	325	42.77	1			1		
Unemployed	123	56.10	1.71	1.13–2.60		1.79	1.14–2.80	
Financial status					< 0.001			0.007
Adequate	257	37.74	1			1		
Inadequate	191	58.12	2.48	1.65–3.71		1.75	1.16–2.64	
Food insecurity (FIES)					0.004			0.037
Secure (0–3)	409	44.25	1			1		
Moderate insecure (≥ 4)	39	69.23	2.87	1.40–5.89		2.25	1.05–4.83	
Perceived social support from family					< 0.001			
Moderate and high (≥ 3)	356	40.73	1					
Low (< 3)	92	68.48	3.16	1.94–5.15				
Perceived social support from friend					0.238			
Moderate and high (≥ 3)	341	44.87	1					
Low (< 3)	107	51.40	1.30	0.84–2.01				
Perceived social support from special person					< 0.001			< 0.001
Moderate and high (≥ 3)	335	38.81	1			1		
Low (< 3)	113	69.03	3.51	2.23–5.54		3.23	2.00–5.21	
Living arrangement					< 0.001			
Family/parents/relative	372	42.74	1					
Friends/work partner/alone/other	76	64.47	2.43	1.46–4.06				
Physical activity					0.838			
Minimally active and HEPA active (≥ 600 MET score)	385	46.23	1					
Inactivity (< 600 MET score)	63	47.62	1.06	0.62–1.80				
Forced displacement					0.001			0.021
No	399	43.61	1			1		
Yes	49	69.39	2.93	1.55–5.56		2.24	1.13–4.40	

Abbreviations: AOR = adjusted odds ratio, CI = confidence interval, COR = crude odds ratio.

### 3.2 Factors Associated With Anxiety Symptoms

In the multivariable logistic regression analysis using a GLMM, several factors remained significantly associated with anxiety symptoms. Only variables meeting inclusion criteria (*p* < 0.25) in univariable analysis were included in the multivariable model. Participants who experienced food insecurity were at a markedly increased risk of anxiety symptoms. Specifically, those with a FIES score of 4 or higher had more than four times the odds of anxiety symptoms compared to food‐secure individuals (AOR: 4.33; 95% CI: 1.99–9.41). Low perceived social support from a special person was another strong predictor of anxiety symptoms. Respondents reporting low levels of such support had nearly three times higher odds of anxiety symptoms compared to those with moderate to high support (AOR: 2.94; 95% CI: 1.68–5.13). Similarly, living arrangement played a critical role; those living alone, with friends or work partners, or in other nonfamily setups had significantly increased odds of anxiety symptoms (AOR: 3.54; 95% CI: 1.94–6.47) compared to those living with family or relatives. Additionally, forced displacement was significantly associated with anxiety symptoms. Individuals who had been displaced had over three times the odds of anxiety symptoms compared to those not displaced (AOR: 3.21; 95% CI: 1.61–6.41) (Table 4).

**Table 4:** Prevalence and factors associated with anxiety symptoms: GLMM (*n* = 448).

Factors	Total	% of anxiety symptoms	COR	95% CI	*p*	AOR	95% CI	*p*
Age					0.669			
18–39	332	20.18	1					
40–59	116	18.10	0.89	0.51–1.53				
Gender					0.518			
Male	186	18.28	1					
Female	262	20.61	1.17	0.73–1.89				
Marital status					0.013			
Married	252	15.48	1					
Single/divorce/widow	196	25.00	1.82	1.14–2.91				
Current employment status					0.850			
Employed	325	19.38	1					
Unemployed	123	20.33	1.05	0.63–1.77				
Financial status					0.040			
Adequate	257	16.73	1					
Inadequate	191	23.56	1.67	1.02–2.74				
Food insecurity (FIES)					< 0.001			< 0.001
Secure (0‐3)	409	17.11	1			1		
Moderate insecure (≥ 4)	39	46.15	4.59	2.27–9.27		4.33	1.99–9.41	
Perceived social support from family					< 0.001			
Moderate and high (≥ 3)	356	15.17	1					
Low (< 3)	92	36.96	3.28	1.96–5.48				
Perceived social support from friend					0.107			
Moderate and high (≥ 3)	341	17.89	1					
Low (< 3)	107	25.23	1.53	0.91–2.58				
Perceived social support from special person					< 0.001			< 0.001
Moderate and high (≥ 3)	335	13.13	1			1		
Low (< 3)	113	38.94	4.22	2.57–6.91		2.94	1.68–5.13	
Living arrangement					< 0.001			< 0.001
Family/parents/relative	372	13.98	1			1		
Friends/work partner/alone/other	76	47.37	5.54	3.24–9.48		3.54	1.94–6.47	
Physical activity					0.552			
Inactivity (< 600 MET score)	63	17.46	1					
Minimally active and HEPA active (≥ 600 MET score)	385	20.00	1.24	0.61–2.53				
Forced displacement					< 0.001			0.001
No	399	16.04	1			1		
Yes	49	48.98	5.02	2.69–9.36		3.21	1.61–6.41	

Abbreviations: AOR = adjusted odds ratio, CI = confidence interval, COR = crude odds ratio.

### 3.3 Factors Associated With Probable PTSD

Only variables meeting inclusion criteria (*p* < 0.25) in univariable analysis were included in the multivariable model. In the adjusted multivariable analysis, living arrangement and forced displacement emerged as significant predictors of probable PTSD among participants. Individuals who lived alone, with friends, work partners, or in other nonfamily settings had significantly higher odds of probable PTSD compared to those living with family, parents, or relatives. Specifically, the odds of experiencing probable PTSD were more than five times higher for individuals in nonfamily living arrangements (AOR: 5.65, 95% CI: 2.80–11.39). Similarly, participants who had been forcibly displaced showed a markedly increased risk of probable PTSD. The odds of probable PTSD among displaced individuals were nearly five times higher than those who had not been displaced (AOR: 4.98, 95% CI: 2.31–10.76) (Table 5).

**Table 5:** Prevalence and factors associated with probable PTSD among the study participants: GLMM (*n* = 448).

Factors	Total	% of probable PTSD	COR	95% CI	*p*	AOR	95% CI	*p*
Age					0.489			
18–39	332	9.94	1					
40–59	116	7.76	0.76	0.35–1.64				
Gender					0.424			
Male	186	8.06	1					
Female	262	10.31	1.31	0.68‐2.54				
Marital status					0.033			
Married	252	6.75	1					
Single/divorce/widow	196	12.76	2.02	1.06–3.86				
Current employment status					0.210			
Employed	325	8.31	1					
Unemployed	123	12.20	1.53	0.79–2.99				
Financial status					0.720			
Adequate	257	8.95	1					
Inadequate	191	9.95	1.12	0.59–2.13				
Food insecurity (FIES)					0.017			
Secure (0–3)	409	8.31	1					
Moderate insecure (≥ 4)	39	20.51	2.91	1.21–7.00				
Perceived social support from family					0.012			
Moderate and high (≥ 3)	356	7.58	1					
Low (< 3)	92	16.30	2.37	1.20–4.68				
Perceived social support from friend					0.713			
Moderate and high (≥ 3)	341	9.09	1					
Low (< 3)	107	10.28	1.15	0.55–2.37				
Perceived social support from special person					< 0.001			
Moderate and high (≥ 3)	335	6.57	1					
Low (< 3)	113	17.70	3.06	1.60–5.85				
Living arrangement					< 0.001			< 0.001
Family/parents/relative	372	5.38	1			1		
Friends/work partner/alone/other	76	28.95	7.17	3.67–14.01		5.65	2.80–11.39	
Physical activity					0.611			
Minimally active and HEPA active (≥ 600 MET score)	385	9.09	1					
Inactivity (< 600 MET score)	63	11.11	1.25	0.53–2.95				
Forced displacement					< 0.001			< 0.001
No	399	6.52	1			1		
Yes	49	32.65	6.96	3.39–14.25		4.98	2.31–10.76	

Abbreviations: AOR = adjusted odds ratio, CI = confidence interval, COR = crude odds ratio.

## 4 Discussion

Our study reveals a significant mental health burden in Eastern Shan State, Myanmar, with depressive symptoms, anxiety symptoms, and probable PTSD affecting 46.4%, 19.6%, and 9.4% of participants, respectively. These figures are notably higher than those reported in other regions. For instance, a nationwide survey conducted during Myanmar's 2021 political crisis found a prevalence of 14.3% for depression, 22.2% for anxiety, and 8.1% for PTSD [18]. Similarly, studies among Myanmar migrant workers in Thailand reported depression rates of 14.4% [19]. In contrast, adolescent populations in Myanmar have shown depression rates of 27.2% and suicidal ideation at 9.4% [20]. Comparing with neighboring countries, a study in Cambodia reported a prevalence of depression at 42.8% and anxiety at 56.0% indicating a higher burden than in Myanmar. However, PTSD prevalence was lower in Cambodia at 2.3% [21]. While this study is grounded in the specific sociopolitical context of Eastern Shan State, the findings are consistent with broader global evidence demonstrating elevated mental health burden in conflict‐affected and resource‐limited settings. Similar patterns of increased depressive symptoms, anxiety symptoms, and probable PTSD have been reported across low‐ and middle‐income countries and humanitarian contexts, highlighting the broader relevance of these findings beyond the local setting.

The elevated rates observed in this study may be attributed to the ongoing conflict, population displacement, and limited access to mental health services in Eastern Shan State, as introduced earlier. These structural challenges likely contribute to chronic stress and reduced coping capacity within affected communities. The higher prevalence of depressive symptoms compared to anxiety symptoms and probable PTSD may reflect the cumulative effects of prolonged socioeconomic hardship and persistent insecurity. These findings highlight the need for context‐specific mental health interventions including improved access to care, strengthened community‐based support systems, and strategies addressing underlying social and economic vulnerabilities. Furthermore, the variation in mental health outcomes across different study settings and countries underscores the importance of context‐specific strategies to effectively mitigate mental health burden and enhance resilience.

### 4.1 Factors Associated With Depressive Symptoms

In this study, the prevalence of depressive symptoms among the study population was significantly associated with multiple social and economic vulnerability factors. Participants reporting inadequate financial status had nearly twice the odds of experiencing depressive symptoms (AOR = 1.75; 95% CI: 1.16–2.64). This finding aligned with prior research including a systematic review of 40 studies that consistently reported strong associations between financial stress particularly subjective economic hardship, debt and poverty and depression across both high‐income and low‐ to middle‐income countries [22]. Similarly, economic instability has been widely documented as a risk factor for depressive symptoms [23]. Unemployment also significantly increased the likelihood of depressive symptoms in our sample (AOR = 1.79; 95% CI: 1.14–2.80), consistent with evidence from previous settings. A study reported that job loss and property damage were strongly linked to elevated depressive symptoms following the Wenchuan earthquake in China [24]. Loss of livelihood often contributes not only to material deprivation but also to a diminished sense of purpose and social identity which are critical components of mental health.

Food insecurity has been consistently associated with depression in previous studies. A nationally representative study of Nepali women of reproductive age, using data from the 2022 Nepal Demographic and Health Survey, found that increasing levels of household food insecurity were significantly associated with higher odds of depression. Women from moderately and severely food‐insecure households had more than triple the odds of depression compared to those who were food secure (odds ratios of approximately 3.07 and 3.01, respectively) [25]. This reinforces the plausibility of our finding that moderate to severe food insecurity doubles the odds of depressive symptoms (AOR = 2.25; 95% CI: 1.05–4.83). From a theoretical standpoint, food insecurity can be viewed as both a chronic stressor and indicator of broader social deprivation, factors linked to poor mental health outcomes in low‐ and middle‐income countries [8].

Low perceived social support from a special person also emerged as a significant factors (AOR = 3.23; 95% CI: 2.00–5.21), consistent with systematic reviews identifying limited social support as a key determinant of depression in conflict‐affected contexts [7]. A longitudinal study among disaster‐affected populations found that perceived emotional support from family and close ties can buffer the negative psychological impact of trauma particularly depressive symptoms [26]. Likewise, forced displacement is a major psychosocial stressor often accompanied by exposure to trauma, loss of social network, and instability. A global review of internally displaced persons and refugees' documents consistently reports high prevalence rates of depression, often ranging between 5% and 80% depending on context and measurement [9]. Although data on forced displacement in similar contexts are limited, our finding that forcibly displaced individuals had over twice the odds of depressive symptoms compared to nondisplaced participants (AOR = 2.24; 95% CI: 1.13–4.40) aligns with international evidence showing that displacement severely undermines mental health. Among conflict‐affected populations like those in Tigray, Ethiopia, depression rates were found to be extremely high (over 80%) illustrating the heavy burden of mental illness among displaced communities [27]. These results underscore the compounding effect of both socioeconomic and psychological vulnerabilities in shaping mental health outcomes.

### 4.2 Factors Associated With Anxiety Symptoms

Anxiety symptoms were significantly associated with multiple social, economic, and psychosocial vulnerabilities in this study. Food insecurity emerged as one of the strongest predictors of anxiety symptoms with affected individuals showing nearly four times the odds of anxiety symptoms (AOR: 4.33; 95% CI: 1.99–9.41). This is consistent with international evidence such as research conducted during the early phase of the COVID‐19 pandemic in the United States, where low food security was associated with markedly increased anxiety risk (ORs ranging from 3.6 to 6.2) [28, 29]. These findings suggest that inadequate access to food may contribute substantially to psychological distress. Participants with low perceived social support from a special person were also at significantly higher risk of anxiety symptoms (AOR: 2.94; 95% CI: 1.68–5.13). This aligned with literature emphasizing the protective role of social support in buffering anxiety and stress responses [7]. Close social relationships may help buffer emotional distress and reduce feeling of isolations.

Living apart from family (e.g., living alone or with friends) was also significantly associated with anxiety symptoms (AOR: 3.54; 95% CI: 1.94–6.47) underlining the role of familial environments in fostering psychological resilience. Research has shown that social isolation and the absence of familiar family structures may be linked to higher anxiety symptoms as individuals face recovery without close relational anchors [30]. Lastly, forced displacement increased anxiety symptoms by more than three‐fold (AOR: 3.21; 95% CI: 1.61–6.41). This is consistent with meta‐analytic studies which document that displacement and residence in temporary shelters significantly elevate the risk of anxiety and PTSD in post‐earthquake populations [31]. Displacement often entails loss of home, disruption of social networks and prolonged instability all of which are potent stressors contributing to anxiety.

### 4.3 Factors Associated With Probable PTSD

PTSD symptoms in this study were strongly associated with multiple adverse experiences related to living without family and forced displacement which were consistent with global patterns observed in disaster‐affected populations. Living without family (e.g., alone or with friends) also significantly increased probable PTSD risk (AOR: 5.65, 95% CI: 2.80–11.39). These results underscored the central protective role of familial support. Prior studies have found that lack of familial or social cohesion is associated with poor PTSD outcomes [32]. Social isolation may limit opportunities for emotional processing and coping, reinforcing feelings of vulnerability. Recent evidence from the 2023 Türkiye–Syria earthquake provides additional support. A cross‐sectional study conducted among non‐direct victims in Turkey, including residents of Istanbul, found that 24% met criteria for PTSD suggesting that even indirect exposure to disaster can have profound psychological effects when combined with displacement, media exposure, or pre‐existing vulnerabilities [33]. Forced displacement emerged as another major predictor (AOR: 4.98, 95% CI: 2.31–10.76). These findings are consistent with previous evidence demonstrating elevated PTSD risk among displaced populations [9, 31]. The physical disruption of displacement often results in secondary stressors (e.g., overcrowding, lack of privacy) that aggravate trauma responses.

Consistent with global evidence, unemployment, food insecurity, and displacement were among the strongest determinants of poor mental health. These factors reflect not only material deprivation but also the erosion of social and cultural anchors that are essential for recovery. The strong association between low perceived social support and both depression and anxiety highlight the protective role of close personal networks in resilience. Conversely, living without family significantly increased the risk of probable PTSD, underscoring the importance of household and kinship structures in mitigating trauma. These results extend the global evidence base by illustrating how mental health outcomes unfold differently in fragile and conflict‐affected settings such as Myanmar, where overlapping crises amplify psychological harm.

Integrating mental health responses should be prioritized through task‐shifting approaches, community‐based psychosocial support, and culturally adapted interventions. Task‐shifting strategies, which involve training nonspecialist health workers to deliver mental health care, have been shown to be effective in low‐resource and conflict‐affected settings [34, 35]. Community‐based psychosocial interventions can enhance resilience and improve access to care in settings where formal mental health services are limited [36]. Furthermore, culturally adapted interventions are essential to ensure acceptability and effectiveness across diverse populations, particularly in fragile and conflict‐affected contexts [7]. These findings have important implications for mental health policy and programming in conflict‐affected regions of Myanmar. Interventions should prioritize integrated mental health services, community‐based psychosocial support, and targeted assistance for displaced and socioeconomically vulnerable populations. Task‐shifting and culturally adapted approaches may improve access to care in resource‐limited settings.

### 4.4 Strength and Limitation of This Study

This study provides important insights into the mental health status of populations in Eastern Shan State, a largely under‐researched and conflict‐affected setting. The use of a relatively large community‐based sample and the inclusion of multiple social, economic, and environmental factors strengthen the comprehensiveness of the analysis. Additionally, the application of established screening instruments and multivariable modeling enhances the analytical rigor of the study. However, several limitations should be considered when interpreting the findings. First, the cross‐sectional design precludes any inference of causal relationships between exposures and mental health outcomes. Second, although previously validated instruments were used, full psychometric and cross‐cultural validation within the local context was not conducted, which may affect measurement validity. Third, the use of screening tools rather than clinical diagnostic assessments means that the findings reflect symptom levels rather than confirmed mental disorders. Furthermore, the study may be subject to selection bias, as institutionalized populations (e.g., hospitalized individuals, prisoners, and military personnel) were excluded. The sample, although adequate for analysis, was drawn from selected townships and may not be fully representative of the broader population of Eastern Shan State or other regions of Myanmar, thereby limiting generalizability. In addition, reliance on self‐reported data may introduce recall bias and social desirability bias, particularly given the stigma surrounding mental health conditions, which may have led to underreporting of symptoms. The findings may not be generalizable beyond similar conflict‐affected settings due to the study's geographic and sampling limitations.

## 5 Conclusions

This study revealed a substantial mental health burden among individuals with nearly one in two experiencing depressive symptoms, one in five reporting anxiety symptoms and one in ten showing signs of probable PTSD. The findings highlighted that trauma exposure, unemployment, food insecurity, forced displacement, low perceived social support, and living apart from family were key factors associated with increased risk of poor mental health. These findings underscore the interconnected social, economic and psychological challenges affecting mental health in this community setting. The results call for integrated mental health support that addresses both emotional distress and underlying structural hardships to strengthen resilience and recovery.

## Author Contributions

**Thein Myint Twin:** conceptualization, data curation, methodology, formal analysis, writing – original draft, writing – review and editing. **Kyaw Min Htike:** conceptualization, data curation, methodology, formal analysis, writing – original draft, writing – review and editing. **Kaung Myat Soe:** conceptualization, formal analysis, writing – original draft, writing – review and editing. **Roshan Kumar Mahato:** conceptualization, methodology, supervision, writing – original draft, writing – review and editing. All authors have read and approved the final version of the manuscript.

## Funding

This research did not receive any specific grant from funding agencies in the public, commercial, or not‐for‐profit sectors. The funding source had no role in study design, data collection, analysis, interpretation, or manuscript preparation.

## Ethics Statement

Ethical approval for this study was obtained from the Centre for Ethics in Human Research, Khon Kaen University, Khon Kaen, Thailand with the reference number of HE 682025.

## Consent

Written informed consent was obtained from all participants prior to data collection. Participation was voluntary, and confidentiality was strictly maintained throughout the study.

## Conflicts of Interest

The authors declare no conflicts of interest.

## Transparency Statement

Roshan Kumar Mahato affirms that this manuscript is an honest, accurate, and transparent account of the study being reported; that no important aspects of the study have been omitted; and that any discrepancies from the study as planned have been explained.

## Supporting information

Supporting File

## Data Availability

Data are available from the corresponding author upon reasonable request. Roshan Kumar Mahato had full access to all data and takes responsibility for the integrity and accuracy of the analysis.
