# Supplementary material for: Burden and Factors Associated With Depressive Symptoms, Anxiety Symptoms, and Probable Post‐Traumatic Stress Disorder in Eastern Shan State, Myanmar: An Analytical Cross‐Sectional Study
Source: Health Sci Rep. 2026 Jun 19;9(6):e72683. doi: 10.1002/hsr2.72683 (PMC13282268; doi:10.1002/hsr2.72683)
Supplement: Supplementary file 1 — Supporting File [file HSR2-9-e72683-s001.docx]

**Supplementary Material 1: Measurement Tools, Scoring Procedures, and Reliability Assessment**

**Measurement of Mental Health Outcomes**

Depression was assessed using the CES-D-10 scale with a cutoff score of ≥10 was used to classify participants as having depressive symptoms (Baron et al., 2017). Anxiety symptom was measured using the GAD-7 scale, a score of ≥8 indicated clinically significant anxiety symptoms (Baker et al., 2018). Probable PTSD was measured using the SPRINT tool, with a cutoff of ≥14 indicating probable PTSD (Norris and Hamblen, 2004). Each of these outcomes was treated as a binary dependent variable (Yes “1” vs No “0” of symptoms). Although these instruments have been widely validated in previous studies, within this study context, only content validity and internal consistency reliability were assessed through expert review and pilot testing. Comprehensive psychometric validation, including construct validity and cultural equivalence testing, was beyond the scope of this study.

**Measurement of Independent Variables**

A structured sociodemographic questionnaire was used to collect information on participants’ characteristics, including age (18–39, 40–59 years), sex (male, female), marital status (married, single/divorced/widowed), and place of residence (urban, rural). Employment status was categorized as employed or unemployed, and financial status was self-reported as adequate or inadequate. Food insecurity was assessed using the Food Insecurity Experience Scale (FIES) and categorized as food secure (scores 0-3) and moderate-to-severe food insecurity (scores ≥4). Perceived social support was measured using the Multidimensional Scale of Perceived Social Support (MSPSS), with scores categorized as low (<3), moderate (3-5), and high (>5) for support from family, friends, and a special person. Living arrangement was categorized as living with family/relatives or non-family living (including living alone, with friends, or with work partners). Physical activity was assessed using metabolic equivalent (MET) scores and categorized as inactive (<600 MET-min/week), minimally active (600-3000 MET-min/week), and health-enhancing physical activity (HEPA) active (>3000 MET-min/week) (Sjostrom et al., 2005). Forced displacement was assessed as a binary variable (Yes/No).

**Scoring and Classification**

Standard scoring procedures were applied for all instruments based on established guidelines. Outcome variables were dichotomised using validated cut-off values. Independent variables were categorised according to standard thresholds or meaningful groupings relevant to the study context.

**Reliability and Validity Assessment**

The instruments were translated into Burmese and Shan languages using forward and backward translation procedures to ensure linguistic and cultural appropriateness. The questionnaires were pre-tested for clarity, cultural relevance, and comprehension. Trained data collectors conducted interviews in private settings to ensure confidentiality and participant comfort. The principal investigator reviewed completed questionnaires daily to ensure completeness and accuracy. Content validity was assessed by three public health experts. A pilot study involving 45 participants was conducted to assess internal consistency reliability using Cronbach’s alpha, yielding acceptable to excellent values: CES-D-10 (α = 0.80), GAD-7 (α = 0.90), SPRINT (α = 0.96), and MSPSS (α = 0.85). Necessary revisions were made based on expert feedback and pilot testing. The instruments used are screening tools and do not provide clinical diagnoses; therefore, outcomes are interpreted as indicative of symptom levels rather than confirmed mental disorders. Participants identified as experiencing severe symptoms or significant psychological distress during interviews were provided with information on available local health services and referred to appropriate healthcare providers where feasible. Data collectors were trained to respond sensitively and to ensure participant safety.

**References**

Baker, A. M., Holbrook, J. T., Yohannes, A. M., Eakin, M. N., Sugar, E. A., Henderson, R. J., Casper, A. S., Kaminsky, D. A., Rea, A. L., Mathews, A. M., Que, L. G., Ramsdell, J. W., Gerald, L. B., Wise, R. A. and Hanania, N. A. (2018), "Test Performance Characteristics of the AIR, GAD-7, and HADS-Anxiety Screening Questionnaires for Anxiety in Chronic Obstructive Pulmonary Disease", *Ann Am Thorac Soc,* Vol. 15 No. 8, pp. 926-934.

Baron, E. C., Davies, T. and Lund, C. (2017), "Validation of the 10-item Centre for Epidemiological Studies Depression Scale (CES-D-10) in Zulu, Xhosa and Afrikaans populations in South Africa", *BMC Psychiatry,* Vol. 17 No. 1, p. 6.

Norris, F. H. and Hamblen, J. L. (2004), "Standardized self-report measures of civilian trauma and PTSD".

Sjostrom, M., Ainsworth, B. E., Bauman, A., Bull, F. C., Hamilton-Craig, C. R. and Sallis, J. F. (2005), "Guidelines for data processing analysis of the International Physical Activity Questionnaire (IPAQ) - Short and long forms", in.
